# Management of Cardiorenal Metabolic Syndrome in Diabetes Mellitus: A Phytotherapeutic Perspective

**DOI:** 10.1155/2014/313718

**Published:** 2014-04-13

**Authors:** Min Kyong Song, Neal M. Davies, Basil D. Roufogalis, Tom Hsun-Wei Huang

**Affiliations:** ^1^The University of Sydney, Faculty of Pharmacy, Sydney, NSW 2006, Australia; ^2^The University of Manitoba, Faculty of Pharmacy, Winnipeg, MB, Canada R3T 2N2; ^3^The University of Sydney, Discipline of Pharmacology, School of Medical Sciences, Sydney Medical School, Sydney, NSW 2006, Australia

## Abstract

Cardiorenal syndrome (CRS) is a complex disease in which the heart and kidney are simultaneously affected and their deleterious declining functions are reinforced in a feedback cycle, with an accelerated progression. Although the coexistence of kidney and heart failure in the same individual carries an extremely bad prognosis, the exact cause of deterioration and the pathophysiological mechanisms underlying the initiation and maintenance of the interaction are complex, multifactorial in nature, and poorly understood. Current therapy includes diuretics, natriuretic hormones, aquaretics (arginine vasopressin antagonists), vasodilators, and inotropes. However, large numbers of patients still develop intractable disease. Moreover, the development of resistance to many standard therapies, such as diuretics and inotropes, has led to an increasing movement toward utilization and development of novel therapies. Herbal and traditional natural medicines may complement or provide an alternative to prevent or delay the progression of CRS. This review provides an analysis of the possible mechanisms and the therapeutic potential of phytotherapeutic medicines for the amelioration of the progression of CRS.

## 1. Introduction 

The appreciation of the interaction between kidneys and heart in disease has led to an increasing biomedical and pharmaceutical interest in recent years [1]. When kidney failure and heart failure coexist, morbidity and mortality are negatively affected [2–4]. Indeed, cardiovascular disease is the leading cause of mortality, consisting of 43.6% of all deaths in patients with end-stage renal disease [5]. Moreover, clinical and epidemiological observations have demonstrated that both kidney failure and heart failure are associated with a high incidence of failure of other organs [6, 7]. The cardiorenal syndrome (CRS) is a complex disease in which both the heart and kidney are simultaneously affected and their deleterious outcomes are reinforced in a feedback cycle, with accelerated progression [8, 9]. One of the most common underlying risk factors for CRS are diabetes and severe atherosclerotic vascular disease [10]. Although the coexistence of kidney and heart failure in the same individual carries an extremely rueful prognosis, the exact causes of deterioration and the pathophysiological mechanisms underlying the initiation and maintenance of the interaction are complex, multifactorial in nature, and poorly understood [11–13]. Plants remain as an important source of therapeutic material for maintaining human health with unparalleled diversity, and they have improved the quality of human life through disease prevention and treatment for centuries [14]. Moreover, medicinal plants are an abundant source of biologically active molecules that play an important role in past and modern medicine which act as a “stepping stone” for the discovery of novel pharmacologically active ligands [15]. Current therapy of CRS includes diuretics, natriuretic hormones, aquaretics (arginine vasopressin antagonists), vasodilators, and inotropes. However, large numbers of patients still develop intractable disease [16]. Moreover, with the development of resistance to many conventional therapies, such as diuretics and inotropes, there is an increasing movement toward novel therapies [17]. This has prompted much interest in the use of traditional medicines for the treatment of CRS. Thus, the current review provides a detailed discussion summarizing the current understanding of herbal and traditional medicines for the management and potential treatment and reversal of CRS-related pathogenesis.

## 2. Cardiorenal Metabolic Syndrome: Current Understanding and Classification

The CRS has been recently defined as a disorder of the heart and kidneys whereby acute or chronic dysfunction in one organ may induce acute or chronic dysfunction in the other [18]. Several groups have proposed that each dysfunctional organ has the* ab initio* ability to initiate and perpetuate disease in the other organ through hemodynamic, neurohormonal, and immunologic/biochemical feedback pathways [11]. Moreover, the current disease definition has been expanded into 5 subtypes that reflect the pathophysiology, time-frame, and bidirectional nature of heart and kidney interactions [19]. Categorizing CRS based on the response to various treatment modalities is practical and ideal in the design of a treatment, including the possibilities of new prevention and management algorithms [20]. Type 1 CRS reflects rapid worsening of cardiac function leading to acute kidney injury. Type 2 CRS comprises chronic abnormalities in cardiac function leading to progressive chronic kidney disease. Type 3 CRS consists of an abrupt worsening of renal function causing acute cardiac dysfunction. Type 4 CRS describes primary chronic kidney disease causing decreased cardiac function, ventricular hypertrophy, diastolic dysfunction, and/or an increased risk of adverse cardiovascular events. Type 5 CRS reflects the presence of combined cardiac and renal dysfunction due to acute or chronic systemic disorders [20, 21].

## 3. Cardiorenal Metabolic Syndrome: Pathophysiology

**Figure 1:**
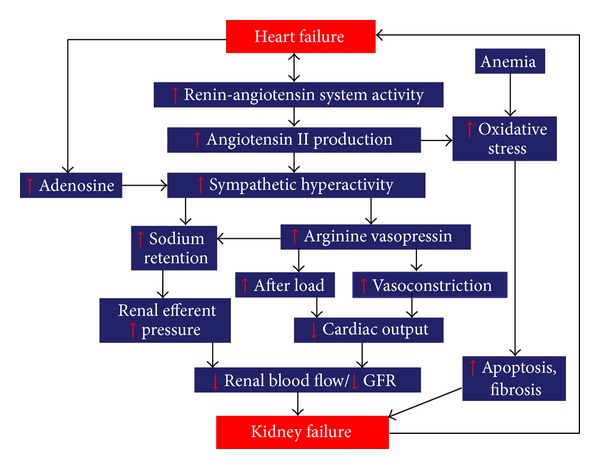
Schematic representation showing the pathophysiological interaction between heart and kidney in CRS and potential sites of intervention by herbal and traditional natural medicine (adapted from [11]). Red arrows indicate the direction of effects of known phytotherapeutic agents.

The pathophysiology of cardiorenal syndrome involves interrelated hemodynamic and neurohormonal mechanisms, including sympathetic overactivity, the renin-angiotensin-aldosterone system, various chemical mediators (nitric oxide, prostaglandins, endothelins, etc.) and oxidative stress [17, 22]. Traditionally, CRS is characterized by an impairment of kidney function, caused by hypoperfusion and cardiac pump function failure [23, 24]. The bidirectional interplay between the heart and the kidneys and the impact of numerous other factors on this interaction have been shown to be fundamental in the pathogenesis of CRS [20] (Figure 1). However, the detailed mechanisms underlying the interplay of CRS still have not been completely delineated [5].

### 3.1. Direct Hemodynamic Mechanisms

The heart and kidneys have been known to share responsibility for maintaining hemodynamic stability through a tight-knit relationship that controls cardiac output, volume status, and vascular tone [25]. CRS is initiated by left ventricular systolic dysfunction, which leads to decreased renal blow flow, followed by activation of fluid retention mechanisms. This subsequently causes worsening of cardiac pumping capacity, resulting in initiating a vicious cycle and eventual organ deterioration [11]. However, the simple hemodynamic variations are only a part of the complex pathophysiology of CRS [1, 20]. The pathophysiology of kidney dysfunction in the context of heart disease is much more complex than a simple reduction of cardiac output [5]. Several other mechanisms are involved and could potentially be considered as a basis for the therapeutic management of this syndrome [20]. The pathophysiology of the cardiorenal syndrome involves interrelated hemodynamic and neurohormonal mechanisms, including the sympathetic nervous system (SNS), the renin-angiotensin-aldosterone system (RAAS), and endothelin and arginine vasopressin system activation [22]. Moreover, neurohormones are strong precipitants and are also mediators of an oxidative injury cascade that can lead to widespread endothelial dysfunction, inflammation, and cell death in the CRS [11].

### 3.2. Autonomic Nervous System

The adverse consequences of sympathetic hyperactivity are one of the harmful compensatory mechanisms that occur in CRS [11]. Sustained elevated adrenergic tone causes a reduction in *β*-adrenergic receptor density, particularly *β*_1_, within the ventricular myocardium, as well as uncoupling of the receptor from the intracellular signalling mechanisms [26]. Less well appreciated are the systemic effects of renal sympathetic stimulation [11]. However, increased kidney sympathetic activation and catecholamine release in the setting of reduced catecholamine clearance with an already impaired kidney function are a part of the self-deteriorating cycle that aggravates kidney dysfunction and heart failure [27].

### 3.3. The Renin-Angiotensin-Aldosterone System

The CRS occurs with both hypoperfusion associated with decreased cardiac output and venous congestion [25]. Downregulation of renal perfusion stimulates renin secretion, which in turn activates the RAAS, followed by activation of the SNS [28]. The extreme sodium avidity and ventricular remodeling conferred by RAAS elaboration in heart failure are a maladaptive response to altered haemodynamics, sympathetic signalling, and progressive renal dysfunction [11]. One of the deleterious actions of the RAAS in the CRS is the activation of nicotinamide adenine dinucleotide phosphate (NADPH) oxidase by angiotensin II (Ang II), resulting in formation of reactive oxygen species (ROS) [29]. Ang II, potentially acting through changes in the cellular redox status, is implicated in vascular inflammation via the nuclear factor kappa B (NF-*κ*B) pathway, which induces production of adhesion molecules [30, 31]. Angiotensin-converting enzyme (ACE) inhibition and aldosterone antagonism have shown a beneficial effect in cardiac failure by inhibiting the intracardiac RAAS, reduction in adrenergic tone, improvement in endothelial function, and prevention of myocardial fibrosis [32]. Moreover, ACE inhibitors and angiotensin receptor blockers have important renoprotective effects in hypertensive patients with nondiabetic renal disease and individuals with diabetic nephropathy [33].

### 3.4. Endothelial Dysfunction

Endothelial dysfunction is one of the major contributors to abnormal vasomotor activity in patients with heart failure [34]. Nitric oxide (NO), an endothelium-derived relaxing factor, is a major regulator of vascular tone through its potent vasodilatory effect [35]. Therefore, deregulation of NO is known to be a major contributor to endothelial dysfunction in heart failure [36]. In addition, the disequilibrium between NO and ROS, by increased ROS production, a low antioxidant status, and lower availability of NO have been shown to increase activity of preganglionic sympathetic neurons. It also stimulates RAAS directly by damaging the renal tubular or intestinal cells or by afferent vasoconstriction with chronic inhibition of NO synthesis [37, 38].

### 3.5. Inflammatory Mediators and Oxidative Injury

The recurrent inflammatory state that is present in both chronic kidney disease and heart failure causes ROS production by activating leukocytes to release the oxidative contents [39]. Ang II has been implicated to be involved in a myriad of inflammatory and oxidative reactions, for instance, infusion of Ang II increased tumour necrosis factor *α* (TNF-*α*) production in the kidney, increased renal synthesis of interleukin (IL)-6, monocyte chemoattractant protein-1 (MCP-1) and elevated tissue levels of activated of NF-*κ*B [40]. Ang II has also been shown to stimulate superoxide generation through activation of the NADH oxidase and NADPH oxidase [29]. Moreover, SNS activity in both kidney and heart failure has been shown to be induced by inflammation through norepinephrine-mediated cytokine production and by releasing neuropeptide Y, which alters cytokine release and immune cell function [5, 41]. Moreover, cytokines have been shown to stimulate renin secretion as a component of the systemic stress response, and tubulointerstitial inflammation has effects on adaptive responses of glomerular hemodynamics and impaired renal function [42].

### 3.6. Arginine Vasopressin

Plasma levels of arginine vasopressin increase in the setting of heart failure. Thus, not only does arginine vasopressin cause vasoconstriction through vasopressin V1 receptors (arteriolar vasoconstriction), and a consequent increase in afterload, but it can also produce water retention via vasopressin V2 receptors (free water reabsorption), which mediate the antidiuretic activity of arginine vasopressin. This combination of effects additionally may enliven the haemodynamic vicious cycle of CRS [43, 44].

### 3.7. Adenosine

The autacoid adenosine is known to have regulatory effects on kidney function through the adenosine A1 receptor. Elevated plasma levels of adenosine have been described in patients with heart failure [45]. Increased adenosine generation has been shown during hypoxia [46], which can occur in patients with heart failure owing to circulatory compromise. Thus, adenosine dysregulation can act as a self-deteriorating cycle synergistically compounding the pathophysiology of CRS.

### 3.8. Cardiorenal-Anaemia Syndrome

Anaemia is common in individuals with chronic kidney and heart disease and has been shown to contribute to an abnormal renal oxidative state [47, 48]. Moreover, it has been shown that severe anaemia could be a causative factor for cardiac and renal disease in patients without previous basic heart disease [39]. Tissue hypoxia as a consequence of anaemia leads to peripheral vasodilatation and decreased vascular resistance, which in turn reduces blood pressure. The SNS is then activated, causing renal vasoconstriction, followed by downregulation of renal blow flow, glomerular filtration rate (GFR), and eventual renal ischemia [49]. The reduced renal blood flow activates the RAAS, causing further vasoconstriction and salt and fluid retention. This fluid retention causes left ventricular hypertrophy, leading to necrosis and apoptosis of myocardial cells, myocardial fibrosis, and cardiomyopathy resulting in heart failure [50, 51]. Anaemia is postulated as a contributor to decreased shear stress, leading to deteriorating cardiac and renal function by various mechanisms including a direct effect of worsening hemodynamic compromise and endothelial dysfunction [52, 53]. Therefore, anaemia has a major role in the pathogenesis of CRS [20].

## 4. Cardiorenal Syndrome in Diabetes

All forms of diabetes are characterized by chronic hyperglycemia and the development of diabetes-specific microvascular pathology in the renal glomerulus, causing nephropathy [54]. Diabetes is also associated with accelerated atherosclerotic macrovascular disease affecting arteries that supply the heart, resulting in coronary heart disease, stroke, peripheral arterial disease, cardiomyopathy, and myocardial infarction [54, 55]. In diabetes, the kidney is involved through progressive sclerosis/fibrosis and proteinuria, due to the overactivity of the transforming growth factor-beta (TGF-*β*) system and the vascular endothelial growth factor (VEGF) system [56]. CRS in diabetes refers to pathophysiological conditions where the heart and the kidneys are simultaneously affected by a systemic disorder leading to injury and/or dysfunction of both organ systems [56].

### 4.1. Diabetic Nephropathy in Cardiorenal Syndrome

Diabetes is a well-established risk factor for cardiovascular disease and a significant proportion of diabetic patients progressively develops clinically significant nephropathy [56]. Diabetic nephropathy (DN) is one of the major complications of diabetes, and a major cause of end stage renal disease in most countries [57–59]. More than 30% of diabetic patients develop clinically evident DN 10 to 20 years from the onset of diabetes mellitus with a 10–30% increase in treatment costs [60]. DN is characterized by excessive amassing of extracellular matrix (ECM), with thickening of glomerular and tubular basement membranes and increased amounts of mesangial matrix, which ultimately progress to glomerulosclerosis and tubule-interstitial fibrosis [61]. Persistent hyperglycemia also activates vasoactive hormonal pathways, including the RAAS and endothelin. These in turn activate second messenger signaling pathways such as protein kinase C (PKC) and MAP kinase (MAPK) and transcription factors such as NF-*κ*B that lead to the alteration in gene expression of growth factors and cytokines such as TGF-*β* [62, 63]. The financial cost of dialysis and the costs of renal transplantation are fiscally prohibitive for patients and health-care systems [64]. Current therapy of DN includes dietary protein restriction, blood pressure control, ACE inhibitors, and angiotensin receptor blockers [65]. However, large numbers of patients still develop intractable disease. This has prompted significant basic and clinical interest in the use of traditional medicines for the treatment of DN [66]. Moreover, these medicines may potentially reverse kidney damage at the onset of proteinuria. However, little is known about the renoprotective effects of herbal medicines [64].

### 4.2. Diabetic Cardiomyopathy in Cardiorenal Syndrome

Diabetic patients have been shown to suffer high mortality rates, with cardiovascular disease being the major cause of death, accounting for some 50% of all diabetes fatalities [56]. Various studies have shown that the risk for cardiovascular events increases by two- to fourfold in patients with type 2 diabetes [67]. Moreover, studies have shown that diabetic patients without previous myocardial infarction have as high a risk of myocardial infarction as nondiabetic patients with previous myocardial infarction [68]. The poor prognosis in diabetic patients with ischemic heart disease has been shown to enhance myocardial dysfunction leading to accelerated heart failure (diabetic cardiomyopathy) [69–71]. Diabetic cardiomyopathy (DC) is characterized by excessive lipid accumulation, with increased triacylglycerol (TAG) stores, and fibrosis in the left ventricle. The known pathogenic mechanisms of DC are metabolic disturbance (depletion of glucose transporter 4, increased free fatty acids, carnitine deficiency, and changes in calcium homeostasis), myocardial fibrosis (associated with increase in Ang II, IGF-I, and inflammatory cytokines), small vessel disease (microangiopathy, impaired coronary flow reserve, and endothelial dysfunction), cardiac autonomic neuropathy (denervation and alterations in myocardial catecholamine levels), and insulin resistance (hyperinsulinemia and reduced insulin sensitivity) [72]. However, all the potential mechanisms have not been completely delineated and no specific treatment combination is presently defined [73]. Therefore, the use of herbal medicine as a therapeutic modality in improving cardiovascular risk has warranted further attention from several researchers [74].

### 4.3. Proteinuria and Cardiorenal Syndrome

Microalbuminuria is a common complication of diabetes and has been a strong predictor of subsequent development of overt DN [75, 76]. Moreover, microalbuminuria is also associated with an increased risk of cardiovascular events and mortality [77]. Studies have shown that diabetic patients with microalbuminuria or proteinuria have a 2–10 times more rapid progression of coronary heart disease, vascular diseases, and arteriosclerosis [78, 79]. In patients with heart failure and renal dysfunction, a new treatment focus has been suggested to first recognize the CRS and treat the whole patient in the long term by optimizing the heart failure therapy while also preserving renal function [56].

## 5. Current Conventional Therapies (Orthodox Medicine)

Orthodox therapeutic management of CRS focuses mainly on correcting hemodynamic abnormalities. However, such an approach is complex and prone to treatment refractoriness and/or worsening dysregulation of one component (e.g., kidney function) by targeting another component (e.g., volume overload) [20]. Although clinical guidelines for managing both heart and kidney diseases have been published, until now agreed-on evidence based clinical treatment guidelines for patients with CRS are lacking [80]. Moreover, with the development of resistance to many standard conventional therapies, such as diuretics and inotropes, there is an increasing interest in developing novel therapies to optimize treatment [17].

## 6. Herbal and Traditional Medicines and Cardiorenal Syndrome

In recent years, there has been growing attention to alternative therapies and the therapeutic use of plant-origin natural products [14]. Herbal medicines have gained significant importance in the last few decades and the demand for use of natural products in the management of cardiovascular and renal diseases [81, 82]. Despite modern pharmacotherapeutics and advancement in an ever-changing world of biotechnology, a lack of understanding still exists with regard to the bioactivity of many phytotherapeutic medicines [83]. This has prompted research to understand the mechanism of action of natural medicines and seek new products for better management of cardiovascular and renal diseases. This section summarizes the current research on various herbal and traditional medicines capable of modulating CRS pathogenesis (Table 1).

### 6.1. Traditional Chinese Medicines

*Apocynum venetum* (Dogbane), traditionally used to calm the liver, soothe the nerves, dissipate heat, and promote diuresis, has shown protective effects on renal function of kidneys of streptozotocin-induced diabetic rats through the modulation of the renal cortex's superoxide dismutase (SOD) and glutathione (GSH) activities [84]. Moreover,* Apocynum venetum* has shown cardiotonic effects through the inhibition of phosphodiesterase 3 (PDE-3) [85]. In addition,* Apocynum venetum* has also been shown to protect cardiac function in the process of ischemia reperfusion through the mechanism of improving energy metabolism, scavenging oxygen free radicals, and inhibiting the production of free radicals in the ischemic myocardium [87].

*Astragalus membranaceus* (*Astragalus*) is a traditional herb used for thousands of years in China and East Asia for kidney disease [118]. Astragalus injection has shown a renal protective effect (i.e., BUN, SCr, CCr, and urine protein) and systemic state improvement (serum albumin level). This study has suggested that although there are unknown bioactive ingredients and an undefined mechanism of renal protection, the role of Astragalus in the treatment of DN may be of clinical significance [119]. Moreover, another experimental study has shown that* Astragalus membranaceus* root is effective in reducing fasting blood glucose and albuminuria levels, in reversing the glomerular hyperfiltration state and in ameliorating the pathological changes of early DN in rat models [86]. Different fractional components isolated from* Astragalus membranaceus* have been shown to protect cardiac function in the process of ischemia reperfusion through the mechanisms of improving energy metabolism, scavenging oxygen free radicals and inhibiting the production of free radicals in the ischemic myocardium [87].

The extract of* Ginkgo biloba* (Ginkgo) leaf has shown protective action on early DN through significantly decreased urinary microalbumin (mALB), alpha1-microglobulin (alpha1-MG), immunoglobulin (IgG), transferrin (TF), retinal binding protein (RBP), and N-acetyl-beta-D-glucosaminidase (NAG) [88].* Ginkgo biloba* extract injection has also been shown to be effective in treating early DN through decreasing urinary albumin excretion rate, regulating blood lipids, improving renal function, and hemorheology [89]. Further study has explored the effect of ginkgo leaf extract on vascular endothelial function in patients with early stage DN.* Ginkgo* leaf extract has been shown to decrease the plasma concentration of Von Willebrand factor (vWF), raise the plasma NO level, and improve the endothelium dependent vascular dilating function in DN patients [90]. Moreover, extract of* Gingko biloba* has been shown to decrease the amounts of serum soluble intercellular cell adhesion molecule-1 (ICAM-1) and soluble vascular cell adhesion molecule-1 (VCAM-1) in patients with early DN [91]. In addition, relevant clinical trials with* Ginkgo biloba* leaves are being carried out, particularly in the treatment of arterial and venous insufficiency and in the prevention of thrombosis. However, the future study of potential benefits of* Ginkgo biloba* in cardiovascular diseases warrants more rigorous systematic investigation of its cardiovascular properties [92].

The root of* Salvia miltiorrhiza*, commonly known as danshen, is traditionally used for treating cardiovascular and inflammatory diseases in East Asian countries [120]. Investigations have shown that* Salvia miltiorrhiza* inhibits the progression of DN by modulating high levels of 24 h urinary protein excretion, the serum and kidney levels TGF-*β*_1_, the kidney concentrations of collagen IV, monocytes/macrophages (ED-1), and the receptor for advanced glycation end-products (RAGE) [93]. Roots of* Salvia miltiorrhiza* have shown protective effects against hypobaric hypoxia through modulation of hypoxia-induced tachycardia, concentration of malonyldialdehyde [94], lipid peroxidase (LPO) and SOD [95]. In addition, danshen has been shown to increase endothelial-dependent vasorelaxation and displayed vasoprotection in ovariectomized (OVX) rats fed with high fat diet, primarily by stimulating NO production, upregulating the mRNA expression of endothelial NO synthase, and downregulating the mRNA expression of TNF-*α*, ICAM-1, and VACM-1 in isolated aortas. These findings indicate that* Salvia miltiorrhiza* is potentially beneficial for preventing cardiovascular disease [96].

*Cordyceps sinensis* (*Cordyceps* Mushroom) is a valued tonic herb to treat a wide range of disorders, including respiratory, renal, liver, and cardiovascular diseases, low libido and impotence, and hyperlipidemia [97].* Cordyceps sinensis* has been shown to ameliorate glomerular sclerosis by reducing proteinuria, decreasing the expressions of fibronectin (FN), collagen-IV, connective tissue growth factor (CTGF), and plasminogen activator inhibitor 1 (PAI-1), and increasing the expression of matrix metalloproteinase-2 (MMP-2) [98]. Further study has shown that* Cordyceps sinensis* affords cardioprotection by reduced postischemic diastolic dysfunction and improved recovery of pressure development and coronary flow. Moreover, this study has also suggested that preischemic adenosine receptor activation may be involved in reducing contracture in hearts pretreated with* Cordyceps sinensis* [99].

### 6.2. Ayurvedic Medicines

*Trigonella foenum-graecum* (Fenugreek) has been reported to possess antidiabetic and antioxidative effects.* Trigonella foenum-graecum* seed aqueous extract has been shown to restore the kidney function of diabetic rats through decreased activities of SOD and catalase, increase concentrations of malondialdehyde in the serum and kidney, and increase levels of 8-hydroxy-2′-deoxyguanosine in urine and renal cortex DNA. Furthermore, all of the ultramorphologic abnormalities in the kidney of diabetic rats, including the uneven thickening of the glomerular base membrane, have been shown to ameliorate by* Trigonella foenum-graecum* treatment [100].* Trigonella foenum-graecum* also has shown a significant decrease in LPO, increase in the activities of key antioxidant enzymes such as SOD, catalase, and glutathione-s-transferase (GST), and reduced GSH contents in heart tissue of diabetic rats [101, 102].

*Terminalia arjuna* (Arjuna) bark, an indigenous plant used in Ayurvedic medicine in India, primarily as a cardiotonic, is also used in treating diabetes, anaemia, tumors, and hypertension [121]. The ethanolic extract of* Terminalia arjuna* stem bark has shown significant reduction in LPO, increase in SOD, catalase, glutathione peroxidase, GST, glutathione reductase and glucose-6-phosphate dehydrogenase, reduced glutathione, vitamin A, vitamin C, vitamin E, total sulfhydryl groups (TSH), and nonprotein sulfhydryl groups (NPSH) in kidney of alloxan-induced diabetic rats [103]. Moreover,* Terminalia arjuna* bark extract has shown a significant prophylactic and therapeutic beneficial effect on protection of heart against isoproterenol-induced chronic heart failure, possibly through maintaining endogenous antioxidant enzyme activities and inhibiting LPO and cytokine levels [104].

*Salacia oblonga* (Ekanayaka) root has been used in the treatment of diabetes and obesity in the Ayurvedic system of Indian traditional medicine [105]. One recent study has shown that SO root attenuates diabetic renal fibrosis, at least in part, by suppressing angiotensin II/AT1 signaling [106]. Chronic administration of* Salacia oblonga* extract has been shown to improve interstitial and perivascular fibrosis through suppression of the overexpression of mRNAs encoding TGF-*β*_1_ and *β*_2_ in the obese Zucker rat heart [107].

Research over the last two decades has revealed that curcumin, one of the active components of* Curcuma longa* (Turmeric), can reverse insulin resistance, hyperglycemia, hyperlipidemia, and other symptoms linked to obesity and obesity-related metabolic diseases [122]. Curcumin has shown protective effects against chronic renal failure by antagonizing TNF-*α*-mediated decrease in PPAR-*γ* and blocked transactivation of NF-*κ*B and repression of PPAR-*γ*. The results have indicated that the anti-inflammatory property of curcumin may be responsible for alleviating chronic renal failure in nephrectomy (Nx) animals [108]. One further study has suggested that curcumin has beneficial effect in the treatment of DC and other cardiovascular disorders, by attenuating myocardial dysfunction, cardiac fibrosis, AGEs accumulation, oxidative stress, inflammation, and apoptosis in the heart of diabetic rats. Moreover, Akt/GSK-3*β* signaling pathway may be involved in mediating these effects [109].

### 6.3. Western Herbal Medicines

*Crataegus oxyacantha* Linn., commonly known as Hawthorn, is one of the most widely used herbal heart tonics [123].* Crataegus oxyacantha* administration has shown a significant attenuation of phosphatase and tensin homolog deleted on chromosome 10 and upregulation of phospho-Akt and c-Raf levels in the heart. This study has suggested that* Crataegus oxyacantha* extract attenuates apoptotic incidence in the experimental myocardial ischemia-reperfusion model by regulating Akt and hypoxia-inducible factor (HIF-1) signaling pathways [110]. Moreover,* Crataegus oxyacantha* has also shown ACE inhibition [111].

Mangiferin, one of the main components of* Mangifera indica* L. (Mango), has been known as a useful cardioprotective agent by reducing oxidative damage [124]. The study has shown that mangiferin inhibits glomerular extracellular matrix expansion and accumulation and TGF-*β*_1_ overexpression in glomeruli of DN rats. Mangiferin was also observed to inhibit the proliferation in high glucose induced-mesangial cells and the overexpression of collagen type IV in AGEs induced-mesangial cells [112]. Moreover, intraperitoneal administration of mangiferin has been shown to exhibit significant decrease in glycosylated haemoglobin and CPK levels, along with the amelioration of STZ-induced oxidative damage, in cardiac tissue and renal tissue [113].

Silymarin, one of the active components of* Silybum marianum* (Milk thistle), is a known antioxidant, hepatoprotectant, and anti-inflammatory agent, with antibacterial, antiallergic, antiviral, and antineoplastic properties [125]. Silymarin has been shown to protect the kidneys against I/R injury through downregulation of increased serum and tissue malondialdehyde, NO, and protein carbonyl [114]. One study has suggested that silymarin has cardioprotective activity against ischemia-reperfusion induced myocardial infarction in rats. Moreover, suppression of the neutrophil infiltration and prevention of the fall in mean arterial pressure and HR during ischemia-reperfusion further support the protection offered by silymarin against ischemia reperfusion injury [115].

*Panax quinquefolius* (North American ginseng) has traditionally been known to be effective on the endocrine, cardiovascular, immune, and central nervous systems [126].* Panax quinquefolius* has shown a preventive effect on DN through downregulation of oxidative stress, NF-*κ*B (p65) levels, ECM proteins, and vasoactive factors [116]. In addition, the effect of Folium* Panax quinquefolius* saponins was conducted on apoptosis of cardiac muscle cells and apoptosis-related gene expression in rats with acute myocardial infarction. This study has shown that Folium* Panax quinquefolius* saponins inhibits cardiac muscle cell apoptosis, downregulates Fas protein expression, upregulates Bcl-2 protein expression, and has antagonistic effect in myocardial ischemic injury [117].

### 6.4. Herbal Toxicology and Safety: Nephropathy

Although the use of Chinese herbal products is increasing, scientific evidence on the safety, efficacy, quality, and regulatory control does not always support such popularity [127]. Some herbal and traditional medicines,containing aristolochic acid, are known to be nephrotoxic and carcinogenic [128]. Aristolochic acid nephropathy (AAN) is characterized by progressive fibrosing interstitial nephritis leading to end-stage renal disease, urothelial malignancy, and severe anaemia [129]. Although botanicals known or suspected to contain aristolochic acid were no longer permitted in many countries, several AAN cases were regularly observed all around the world [130]. Moreover, medicinal herbal extracts may exert renal toxicity through their inherent properties, making it important to continue compiling information regarding the potential toxicity of all medicinal herbs [131]. The toxicology and safety of flavonoids prepared using local available botanicals also demonstrated cases of acute renal failure after use of* Taxus celebica*, ciandidaol, and* Cupresssus funebris* [132]. Toxicological effects of herbal use could also have potential detrimental effects on the CRS and this also needs to be researched and characterized clinically and mechanistically.

## 7. Conclusion

The association between kidney failure and cardiovascular diseases has been shown repeatedly, particularly in the past decade [133–135]. CRS is an interdependent involvement of both the heart and the kidney, which can progress in a spiraling fashion, leading to volume overload, diuretic resistance, and further involvement of all organ systems in which the clinical condition will likely worsen and multiorgan system failure can ensue [5]. Moreover, clinical trials have shown that deterioration of renal function decreases after the first myocardial infarction, particularly in patients who already had impairment of renal function [136, 137]. The heterogeneous and complex pathophysiology of CRS makes patient management an intricate clinical challenge [22]. Although clinical guidelines for managing both heart failure and chronic renal disease have been drawn, until now agreed-upon guidelines surrounding the therapy of patients with CRS are lacking. Therefore, future treatment directions should take into consideration both kidney and heart function [80]. The current orthodox pharmaceutical treatments, such as diuretics, vasodilators, or inotropes, could cause a reduction in plasma volume, renal perfusion redistribution with cortical vasoconstriction, decrease in preload with an increase in venous congestion, and further neurohormonal activation, leading to a worsening outcome [80]. Moreover, the increasing importance of understanding the specific molecular and biochemical changes in CRS emphasizes the requirement for development of novel therapeutic interventions. Herbal medicine has a long and respected history and holds a valuable place in the treatment of cardiovascular and kidney diseases [138, 139]. This review confirms that natural and traditional herbal medicines have potential as alternative or combination (complementary) therapy for CRS. Despite the long history of herbal and natural traditional medicines for the management of CRS, there is still no conclusive evidence for their effectiveness or their safety profiles. Therefore, further investigation into their exact mechanisms of action are warranted and required to gather proof of efficacy and safety for possible protection against CRS-related pathophysiology and disease progression.

## Figures and Tables

**Table 1:** Modern research on natural medicines capable of modulating cardiorenal syndrome related pathogenesis.

Herbal medicines	Functions	References
*Apocynum venetum* (Dogbane)	Modulating effect on SOD and GSH activities. Improving energy metabolism, scavenging oxygen free radicals, and inhibition of PDE-3	[84, 85]

*Astragalus membranaceus* (*Astragalus*)	Modulating effect on BUN, SCr, CCr, urine protein, and serum albumin levels. Reducing fasting blood glucose, albuminuria levels, and reversing the glomerular hyperfiltration state	[86, 87]

*Ginkgo biloba* (Ginkgo)	Downregulating the levels of urinary mALB, alpha1-MG, IgG, TF, RBP, and NAG. Decreasing the levels of vWF, ICAM-1, and VCAM-1. Raise the plasma NO level and improve the endothelium dependent vascular dilating function	[88–92]

Root of *Salvia miltiorrhiza* (Danshen)	Modulating effect on levels of TGF-*β*_1_, collagen IV, ED-1, RAGE, malondialdehyde, LPO and SOD. Upregulating the level of endothelial NO synthase and downregulating the levels of TNF-*α*, ICAM-1, and VACM-1	[93–96]

*Cordyceps sinensis* (*Cordyceps* Mushroom)	Decreasing the levels of FN, collagen-IV, CTGF and PAI-1, and proteinuria and increasing the level of MMP-2	[97–99]

*Trigonella foenum-graecum* (Fenugreek)	Increasing concentrations of malondialdehyde and level of 8-hydroxy-2′-deoxyguanosine. Decreasing the levels of LPO and increasing the levels of CAT, GST, and GSH	[100–102]

*Terminalia arjuna *stem bark (Arjuna)	Downregulation in LPO. Upregulating the levels of SOD, catalase, GSH peroxidase, GST, GSH reductase and glucose-6-phosphate dehydrogenase, GSH, and total TSH and NPSH	[103, 104]

*Salacia oblonga*(Ekanayaka)	Suppressing angiotensin II/AT1 signaling and overexpression of TGF-*β*_1_ and *β*_2_	[105–107]

Curcumin from *Curcuma longa*(Turmeric)	Antagonizing TNF-*α*-mediated decrease in PPAR-*γ* and blocked transactivation of NF-*κ*B and repression of PPAR-*γ*. Attenuating myocardial dysfunction through Akt/GSK-3*β* signaling pathway	[108, 109]

*Crataegus oxyacantha* Linn. (Hawthorn)	Attenuating apoptotic incidence by regulating Akt and HIF-1 signaling pathways. Significant attenuation of phosphatase and tensin homolog deleted on chromosome 10 and upregulation of phospho-Akt and c-Raf levels. ACE inhibiting effect	[110, 111]

Mangiferin from *Mangifera indica* L. (Mango)	Inhibition of glomerular ECM expansion and the levels of TGF-*β*_1_ and collagen IV. Decrease in glycosylated hemoglobin and CPK levels	[112, 113]

Silymarin from *Silybum marianum* (Milk Thistle)	Downregulating the levels of malondialdehyde, NO, and protein carbonyl. Suppression of the neutrophil infiltration and preventing the fall in mean arterial pressure and HR during ischemia-reperfusion	[114, 115]

*Panax quinquefolius * (North American ginseng)	Downregulating the levels of NF-*κ*B (p65), ECM proteins, vasoactive factors, and Fas. Upregulating the level of Bcl-2	[116, 117]
